# A new era in meningitis prevention: implications of Nigeria’s 5-in-1 vaccine rollout

**DOI:** 10.1097/MS9.0000000000002199

**Published:** 2024-05-20

**Authors:** Ayush Anand, Amogh Verma, Nathnael A. Woldehana, Prakasini Satapathy, Rakesh K. Sharma, Divya Sharma, Mithhil Arora, Mahalaqua N. Khatib, Shilpa Gaidhane, Quazi S. Zahiruddin, Sarvesh Rustagi

**Affiliations:** a B.P. Koirala Institute of Health Sciences, Dharan, Nepal; b MediSurg Research, Darbhanga; c Rama Medical College Hospital and Research Centre, Hapur; d Center for Global Health Research, Saveetha Medical College and Hospital, Saveetha Institute of Medical and Technical Sciences, Saveetha University, Chennai; e Division of Evidence Synthesis; f South Asia Infant Feeding Research Network (SAIFRN), Division of Evidence Synthesis, Global Consortium of Public Health and Research; g One Health Centre (COHERD), Jawaharlal Nehru Medical College, Datta Meghe Institute of Higher Education, Wardha; h Graphic Era (Deemed to be University); i Graphic Era Hill University, Clement Town; j School of Applied and Life Sciences, Uttaranchal University, Dehradun, Uttarakhand; k Chitkara Centre for Research and Development, Chitkara University, Himachal Pradesh; l Centre of Research Impact and Outcome, Chitkara University, Rajpura, Punjab, India; m Myungsunng Medical College, Addis Ababa, Ethiopia; n Johns Hopkins School of Public Health, Baltimore, Maryland, USA; o Medical Laboratories Techniques Department, Al-Mustaqbal University, Hillah, Babil, Iraq

Meningitis, a severe inflammation of the brain and spinal cord membranes, has long stood as a formidable public health challenge, particularly in the African Meningitis Belt^1^. However, the recent introduction of the Men5CV vaccine by Nigeria covers five major strains of this bacterium and marks a revolutionary step in the battle against this deadly disease. Nigeria is the first country to introduce this vaccine. The rollout of MenFive in Nigeria is a testament to what can be achieved with international cooperation in vaccine development and public health. It exemplifies the critical role of global health partnerships and funding in tackling infectious diseases, particularly in low-resource settings.

The Men5CV vaccine, also branded as MenFive, is the culmination of 13 years of rigorous research and collaboration between PATH (Program for Appropriate Technology in Health) and the Serum Institute of India, with significant backing from the UK government’s Foreign, Commonwealth and Development Office. This vaccine is unique because it offers comprehensive protection against the five strains of the meningococcus bacteria – A, C, W, Y, and X – most prevalent in the African meningitis belt^1–3^. This is a considerable advancement over existing vaccines, which primarily offer protection against only one strain. The technology behind MenFive draws on the successes of the MenAfriVac vaccine, which has virtually eliminated epidemics caused by the meningococcus A strain in sub-Saharan Africa^2^. The extension of this technology to cover multiple strains can potentially transform the landscape of meningitis prevention, providing a broader shield against this unpredictable and often fatal disease.

The introduction of MenFive in Nigeria – amid an outbreak that resulted in over 1700 suspected cases and 153 deaths – underscores the vaccine’s timely necessity^2^. This rollout not only aims to curb the current outbreak but also sets a precedent for rapid response vaccination campaigns against multiple strains of meningitis. The success in Nigeria could lead to wider adoption across the African continent, offering hope for a substantial reduction in incidence and mortality from the disease. Meningitis outbreaks not only cause significant mortality and morbidity but also place a heavy economic burden on African nations due to healthcare costs and lost productivity^4^. By potentially reducing the frequency and severity of outbreaks, MenFive can contribute to economic stability and growth. Socially, the vaccine can alleviate some of the fear and disruption that recurrent meningitis outbreaks cause. Schools, markets, and social gatherings can proceed without the pervasive fear of infection, fostering a stronger sense of community and normalcy. This will ensure a higher quality of life in Africa.

Effective use of MenFive requires robust health systems to manage logistics, from cold chain maintenance to the administration of vaccines in remote areas. Though prior studies have demonstrated non-inferiority to established vaccines and adequate immunogenicity, exact data regarding efficacy is unknown. Hence, ongoing surveillance to monitor vaccine efficacy and the emergence of any non-covered strains is mandated. Also, scaling up production, securing sustained funding, and ensuring equitable distribution remain formidable challenges. The vaccine’s deployment through the emergency stockpile managed by the International Coordinating Group on Vaccine Provision is a temporary measure. A long-term strategy requires integration into national immunization programs supported by international donors and local governments.

In conclusion, the introduction of the Men5CV vaccine by Nigeria is a landmark event in the fight against meningitis. It holds the promise of saving millions of lives and fundamentally changing the course of health in Africa and beyond. As this vaccine begins its journey from a hopeful beginning to a widespread tool of health empowerment, the global health community must remain committed to supporting and expanding its reach. The WHO’s goal to eliminate meningitis as a public health problem by 2030 is ambitious. Achieving it will require not only vaccines like MenFive but also a sustained commitment to health system strengthening, community engagement, and global health security.

## Ethical approval

Ethical approval is not applicable for this editorial article.

## Consent

Informed consent is not applicable for this editorial article.

## Sources of funding

Not applicable.

## Author contribution

A.A.: conceptualization, project administration, supervision, validation, visualization, writing – original draft, and writing – review and editing; A.V.: visualization, writing – original draft, and writing – review and editing; N.A.W.: project administration, validation, visualization, writing – original draft, and writing – review and editing; P.S., R.K.S., D.S., M.A., M.N.K., S.G., Q.S.Z., and S.R.: supervision, validation, and writing – review and editing.

## Conflicts of interest disclosure

The authors declared no conflicts of interest.

## Research registration unique identifying number (UIN)

Not applicable.

## Guarantor

Ayush Anand.

## Data availability statement

Not applicable.

## Provenance and peer review

Not commissioned, externally peer-reviewed.
